# Optimal Contrast Agent Staining of Ligaments and Tendons for X-Ray Computed Tomography

**DOI:** 10.1371/journal.pone.0153552

**Published:** 2016-04-14

**Authors:** Richard Balint, Tristan Lowe, Tom Shearer

**Affiliations:** 1 School of Materials, University of Manchester, Manchester, United Kingdom; 2 Henry Moseley X-ray Imaging Facility, School of Materials, University of Manchester, Manchester, United Kingdom; 3 School of Mathematics, University of Manchester, Manchester, United Kingdom; Pennsylvania State Hershey College of Medicine, UNITED STATES

## Abstract

X-ray computed tomography has become an important tool for studying the microstructures of biological soft tissues, such as ligaments and tendons. Due to the low X-ray attenuation of such tissues, chemical contrast agents are often necessary to enhance contrast during scanning. In this article, the effects of using three different contrast agents—iodine potassium iodide solution, phosphotungstic acid and phosphomolybdic acid—are evaluated and compared. Porcine anterior cruciate ligaments, patellar tendons, medial collateral ligaments and lateral collateral ligaments were used as the basis of the study. Three samples of each of the four ligament/tendon types were each assigned a different contrast agent (giving a total of twelve samples), and the progression of that agent through the tissue was monitored by performing a scan every day for a total period of five days (giving a total of sixty scans). Since the samples were unstained on day one, they had been stained for a total of four days by the time of the final scans. The relative contrast enhancement and tissue deformation were measured. It was observed that the iodine potassium iodide solution penetrated the samples fastest and caused the least sample shrinkage on average (although significant deformation was observed by the time of the final scans), whereas the phosphomolybdic acid caused the greatest sample shrinkage. Equations describing the observed behaviour of the contrast agents, which can be used to predict optimal staining times for ligament and tendon X-ray computed tomography, are presented.

## Introduction

X-ray computed tomography (XCT) is an important tool for developing an understanding of the mechanical properties of soft tissues. XCT-generated three-dimensional (3D) images are used to measure key parameters, such as the collagen volume fraction and fibre alignment direction, that are required for the creation of mathematical models that describe soft tissue behaviour [1, 2]. Whilst 3D images can be obtained by layering scanning electron microscope [3] or histology [4] images, it is possible that artefacts will be induced due to the release of residual stress when the samples are cut into the thin slices that are necessary for such techniques. The aorta, for example, is known to open out into an open sector when sliced [5]. This is, of course, a considerably different geometry to the annular cylindrical shape of the aorta’s natural *in vivo* configuration. XCT, on the other hand, is a rapid method that does not require the slicing of samples. Therefore, it allows 3D images of tissues in a configuration much closer to their natural state to be obtained.

A major difficulty in obtaining useful XCT images of soft tissues is the low contrast they exhibit due to their low X-ray attenuation. This can make distinguishing between two different soft tissue types challenging. In order to overcome this difficulty, several authors have utilised contrast agents, which ideally bind to a specific tissue and therefore increase the contrast between it and its environment. Metscher [6,7] used iodine potassium iodide (I2KI) solution and phosphotungstic acid (PTA) to enhance contrast in several types of sample, including vertebrates, embryos, insects and other invertebrates. Jeffery *et al*. [8] were able to determine the alignment of muscle fascicles in rodent heads by using I2KI staining. I_2_KI was also used by Stephenson *et al*. [9] and Aslanidi *et al*. [10] to enhance contrast in rat and rabbit hearts, by de Crespigny *et al*. [11] to visualise mouse brains and by Wong *et al*. [12] to image mouse embryos. Pauwels *et al*. [13] investigated the diffusion of 28 different contrast agents into porcine adipose and muscle tissue, and mouse legs. Their study concluded that potassium iodide (KI) and sodium tungstate diffuse into soft tissue samples most efficiently, whereas PTA, phosphomolybdic acid (PMA) and Mercury (II) chloride provide the best contrast between different soft tissue types.

A key consideration when staining samples is the incubation time. Both over-staining and under-staining can lead to undesirable results. If a sample is incubated for too short a period, the stain will not be able to perfuse into it completely, and therefore the tissue will retain low contrast in its centre [14]. Over-staining, however, can lead to sample deformation and tissue shrinkage [15]. Thus, it is desirable to determine optimal incubation times, which depend both on tissue type and the contrast agent being used. Butters *et al*. [16] investigated the optimal I2KI staining of rat hearts, and Shearer *et al*. [14] investigated the optimal I2KI and PTA staining of porcine anterior cruciate ligaments (ACLs) and patellar tendons (PTs) using low concentration contrast agents. In the latter study the contrast agents did not diffuse into the centres of the samples even after 150h of staining, which was likely due to their low (0.3% w/v) concentration. Therefore, stains were used at a much higher (10% w/v) concentration in the present work.

To the authors’ knowledge, there are two existing studies which utilise micro-XCT to quantitatively measure aspects of whole ligament or tendon microstructure [14,17]. In both studies, the resulting 3D images were used to track fascicle alignment through the length of the samples. Whilst Kalson *et al*. [17] were able to achieve this without using contrast agents, this came at the expense of scanning time, which was 12 hours in that study. The use of contrast agents allows a significant reduction in scanning time (approximately 24 minutes in [14]). If scanning time can be further reduced, for example by using a synchrotron as opposed to a laboratory-based X-ray source and/or by taking fewer projections, it may be possible to use contrast-enhanced XCT to observe how fascicle alignment changes in real time as a ligament or tendon is stretched or subjected to torsion. To do so effectively, however, it is necessary to develop an optimal staining protocol. In order to enable automated segmentation of XCT images, it is desirable to achieve a consistent intensity profile throughout the depth of a sample; therefore, it is of interest to know how intensity at a given depth changes through time as stain diffusion takes place.

In previous studies, dissected samples were maintained at their *in vivo* length; for example, by suturing them to a plastic rod [17], or by gluing their ends to discs that were held apart by a fixed distance [14]; however, when scanning soft tissues with the aim of mathematically modelling them, it is important that they are kept as closely as possible to their stress-free configurations since the presence of stresses can lead to modelling inaccuracies [18]. These stresses may also affect the surface area of the sample, and hence modify the time it takes for a contrast agent to diffuse into it. In this study, therefore, the samples were not kept at their *in vivo* lengths, but were instead allowed to hang freely under their own weight, thus removing any tension that may have been present.

Ligaments and tendons have a complex hierarchical structure consisting of subunits across many different length scales. From largest to smallest, these are: fascicles (with a diameter of 50–400 μm), fibres (10–50 μm), fibrils (50–500 nm), sub-fibrils (10–20 nm), microfibrils (3.5 nm) and finally, the tropocollagen molecule (1.5 nm) [19,20]. The arrangement of these subunits, which are embedded in an extracollagenous matrix, varies between different types of ligament and results in fascicles of considerably different sizes and shapes [21,22]. XCT can be used to determine how fascicle morphology varies, not only in cross-section, but in 3D and could potentially be used to measure the collagen volume fraction of a given sample.

In this paper, the ability of I2KI, PTA and PMA to enhance contrast in the XCT of knee ligaments and tendons is investigated. It is shown that I2KI diffused into the samples most quickly and caused the least sample shrinkage, whereas PMA diffused into the samples the slowest and caused the most sample shrinkage.

## Materials and Methods

### Ethics Statement

The ligaments used in this study were from pig legs purchased from Kurpas Meats PLC (location coordinates: [53.471697,-2.175908]). The pigs were slaughtered at the John Penny and Sons slaughterhouse (location coordinates: [53.849531,-1.678699]) according to United Kingdom slaughter laws. The animals were not slaughtered specifically for research, and the ligaments/tendons are a by-product of the meat industry; therefore, ethical approval was not required.

### Sample preparation

The ACLs, PTs, medial collateral ligaments (MCLs) and lateral collateral ligaments (LCLs) were dissected from three pig legs with their bone attachments still in place within 48 hours of slaughter, giving a total of twelve samples. The upper bone attachment of each sample was glued to the aluminium lid of a polystyrene container (source: Starlab (UK), Ltd, Milton Keynes, UK) using Loctite 435 (source: Loctite UK & Ireland, Hemel Hempstead, UK). The sample was then allowed to hang freely under its own weight in order to be as close as possible to its stress-free configuration. All samples were fixed in 10% v/v formalin solution, and stored in that solution until the first day of scanning. Three different contrast agents were prepared: 10% w/v I2KI solution [3.3% w/v iodine metal (I_2_) + 6.7% w/v KI in water], 10% w/v PTA solution and 10% w/v PMA solution. One sample of each tissue type was allocated to be stained with each contrast agent. This was done to avoid bias due to the different sizes and collagen contents of the four types of ligament used in this study [23].

The samples were scanned once per day over a period of five days, with staining taking place overnight in between each scan. Therefore, the samples were unstained for their first scan on day one, and had been stained for four days by the time of their final scan on day five. The samples were removed from the contrast agent only for the duration of the scan, during which they were stored in the polystyrene containers mentioned above. A small reservoir of phosphate-buffered saline solution was added to the bottom of the containers in order to saturate the humidity within them, and therefore minimise sample shrinkage due to drying during scanning. To maintain the required contrast agent concentration, the staining solution was changed after each scan.

### Scanning procedure

The samples were imaged using a Nikon Metris XT H 225 system with an amorphous silicon 14bit PaxScan(R)4030E detector at the Henry Moseley X-ray Imaging Facility at The University of Manchester. The source-to-specimen and source-to-detector distances were 57.3 mm and 965 mm, respectively. The samples were placed on the rotation stage and were scanned with a tungsten source, with an accelerating voltage of 60 kV, a current of 280 μA and a gain of 30 dB. A total of 2001 projections was taken over 360° with an exposure time of 708 ms/projection, giving a total acquisition time of just under 24 minutes. The resolution obtained was 7.5 μm per voxel.

### Analysis

After image acquisition, data sets were reconstructed using the Nikon Metris CT-Pro reconstruction software (Metris XT2.2, Service Pack 10). No beam-hardening correction [24] or noise reduction was applied to ensure that the grey-level intensity of each image was not artificially modified, and therefore was the result of the diffusion of the contrast agents alone.

**Fig 1:**
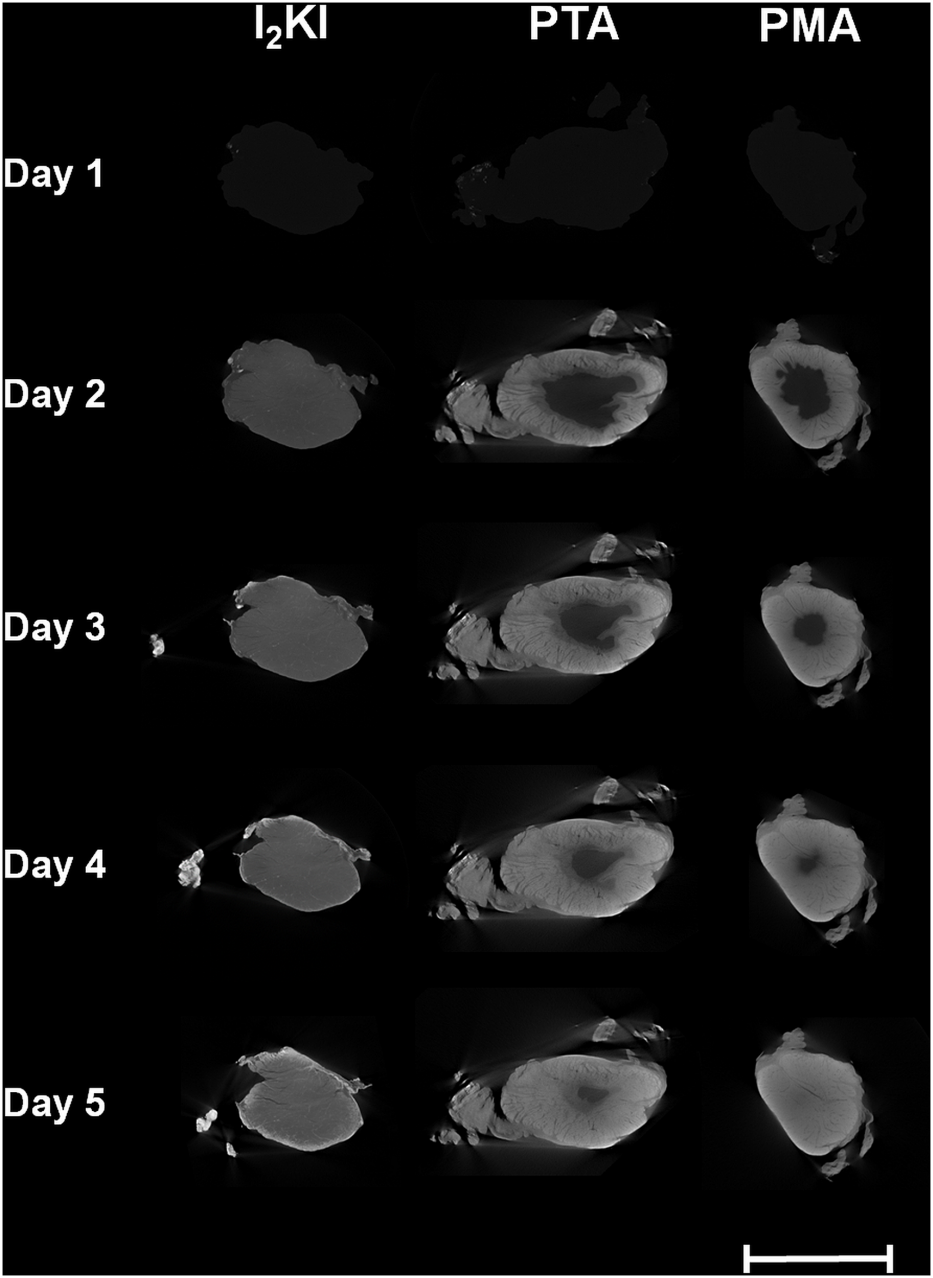
Reconstructed virtual XCT transverse slices of three porcine ACL samples. Each sample was stained with a different contrast agent (I2KI, PTA and PMA) for 0, 1, 2, 3 and 4 days. Scale bar = 10 mm.

**Fig 2:**
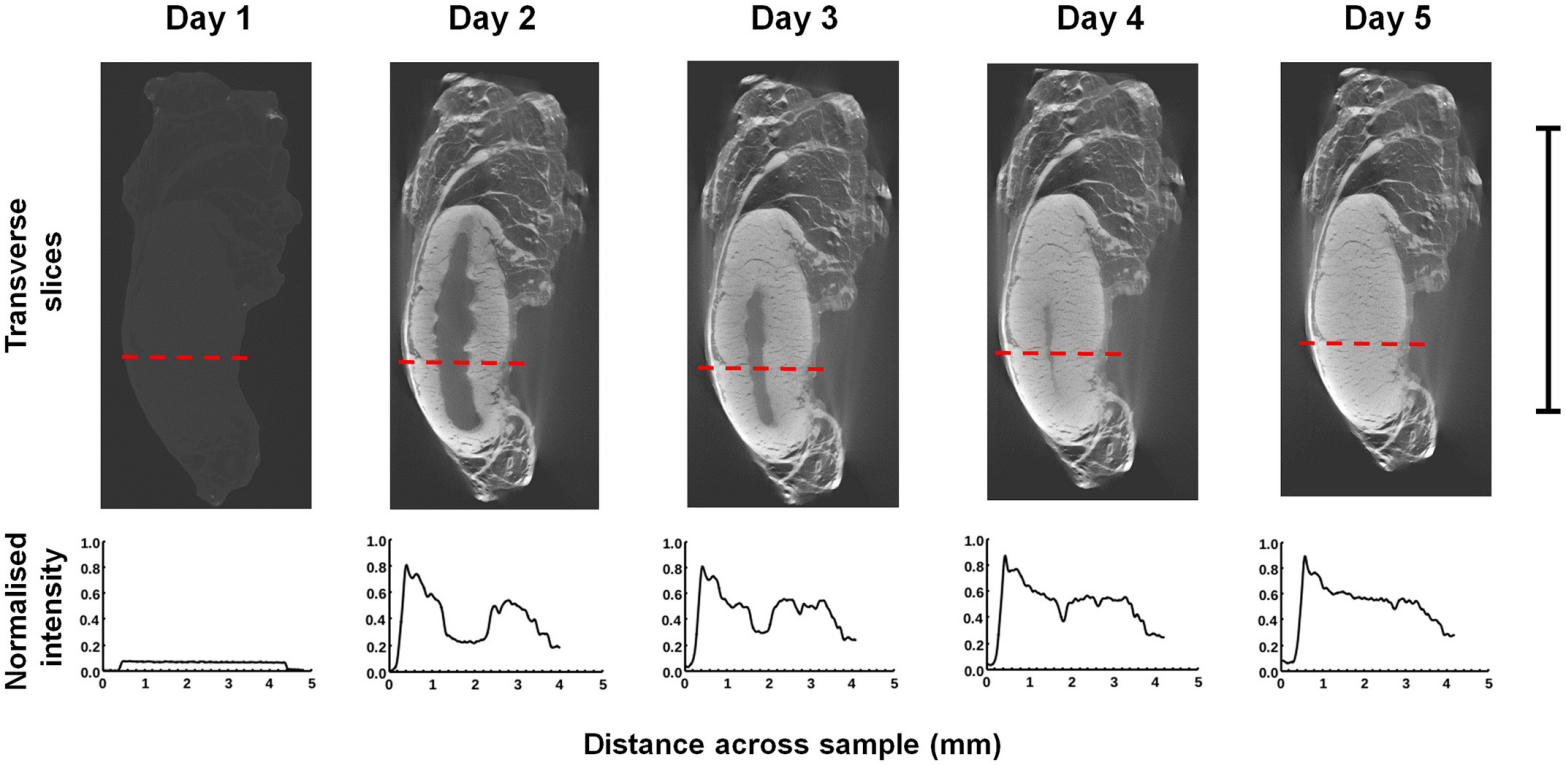
Transverse slices and normalised intensity line profiles for the PTA-stained LCL after 0, 1, 2, 3 and 4 days of staining. The red dashes on the transverse slices indicate the paths of the lines that were profiled. Scale bar = 10 mm.

**Fig 3:**
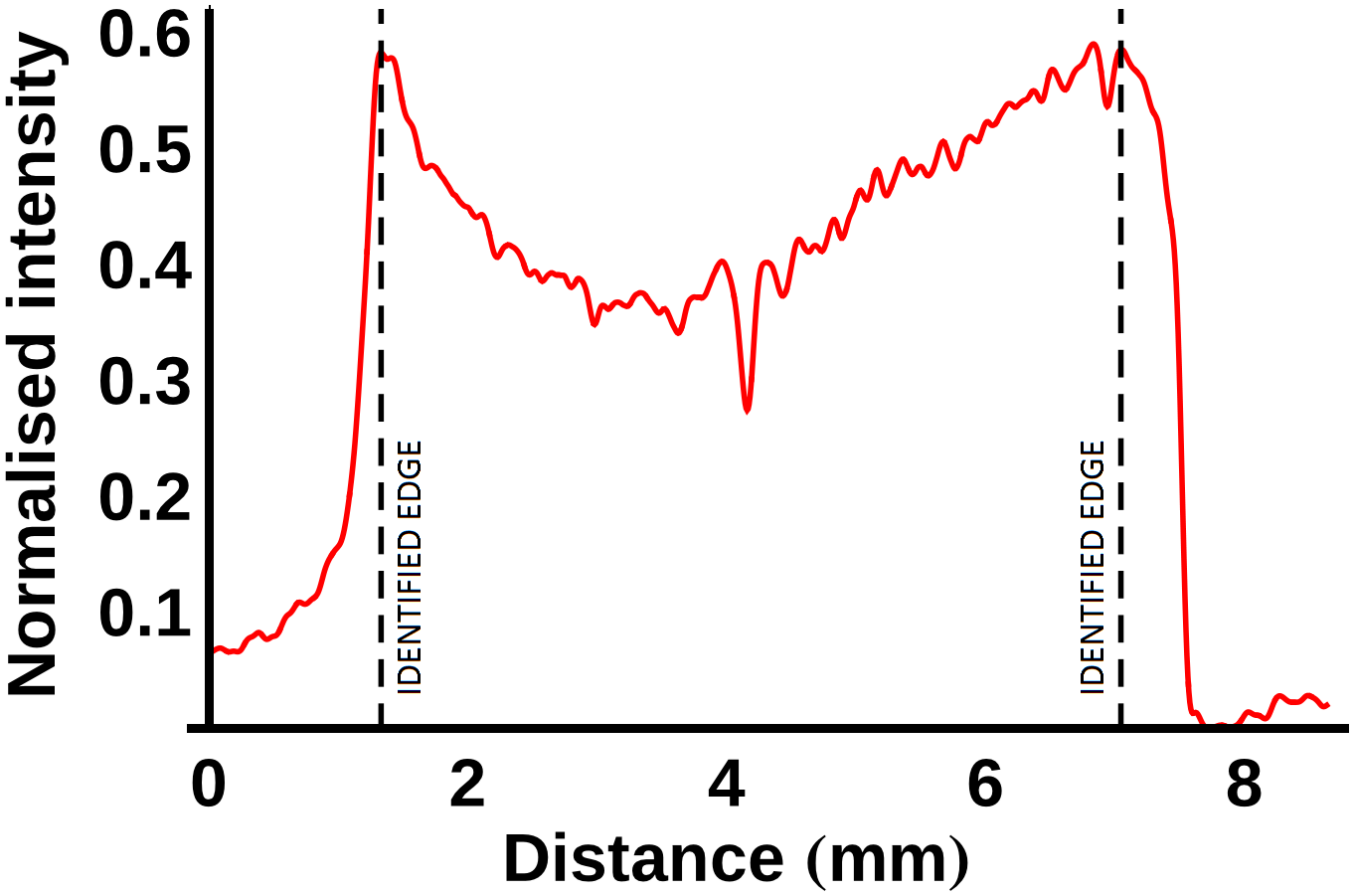
Method adopted for detecting the sample edges. First the normalised intensity line profile is filtered with a Gaussian kernel with σ = 4 (red line) in order to reduce noise. The maximum (minimum) intensity gradient was detected and the sample edge (dashed, black line) was identified as the next point towards the sample centre at which the absolute value of the intensity gradient was approximately zero (<0.01).

Following reconstruction, two-dimensional virtual transverse slices were extracted midway through each ligament and tendon (see Fig 1). A line profile was plotted through the centre of each slice with a width of 10 pixels. As an example, the line profiles of the PTA-stained LCL are plotted in Fig 2 (note that the vertical axis in this figure shows the *normalised* intensity; this value is obtained by dividing the observed intensity in a given line profile by the maximum intensity that was recorded across any of the sixty line profiles that were plotted, thus giving a number between 0 and 1). The ligament edge was identified using a three-stage process based on that outlined in [16]: 1) the line profile was filtered with a Gaussian kernel (σ = 4), 2) the maximum intensity gradient was located, and 3) the edge was defined as the next point towards the sample centre where the absolute value of the intensity gradient was approximately zero (<0.01). The opposing edge was identified as the last point from the sample centre where the absolute value of the intensity gradient was approximately zero before the location of the minimum gradient. This process is illustrated for the PMA-stained ACL (after four days of staining) in Fig 3. Finally, to remove the effects of short length scale sample variability, every point in each sample was assigned an intensity value equal to the average intensity within a 0.1mm neighbourhood of that point.

**Fig 4:**
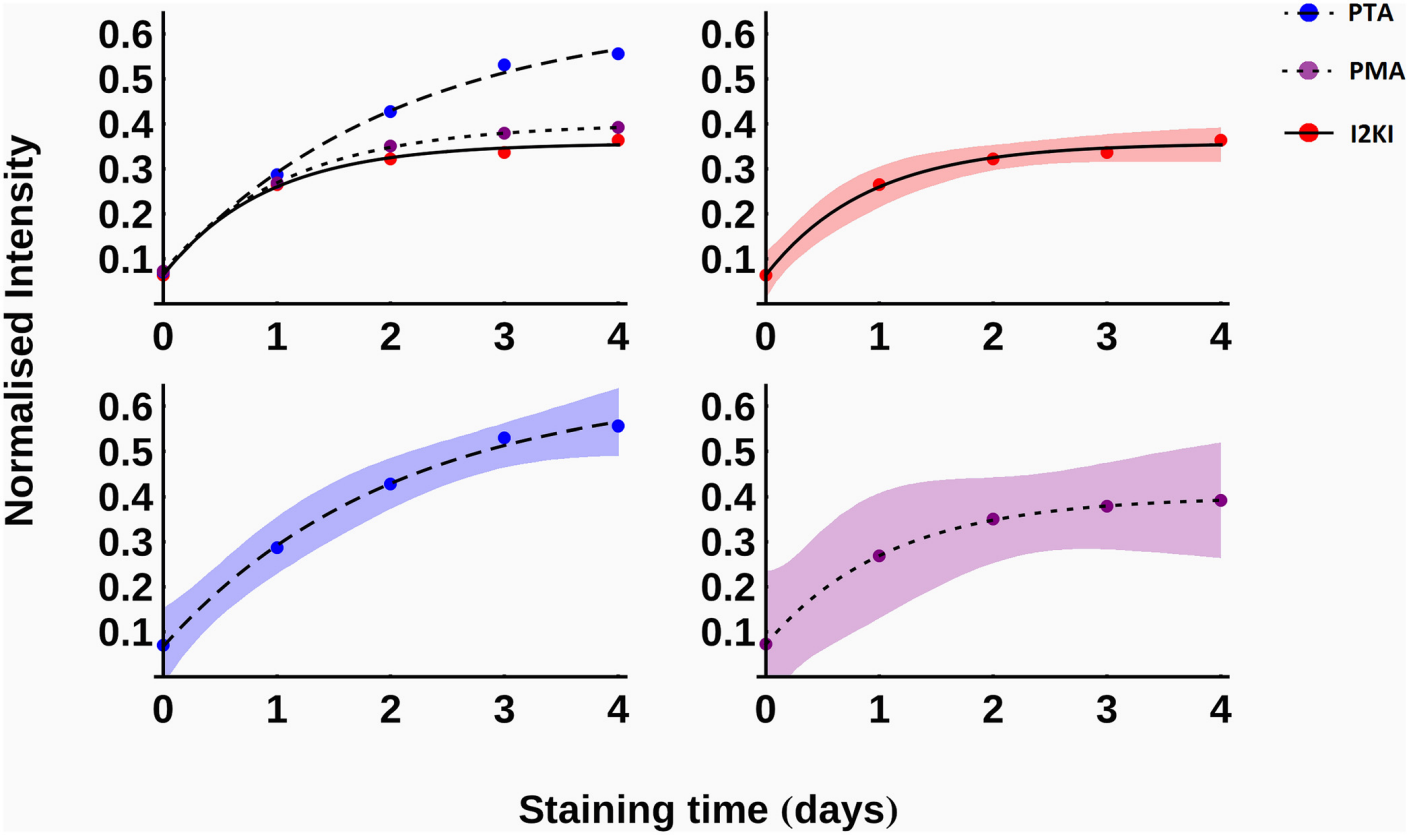
The average, normalised intensity at a depth of 1mm into the samples as a function of time. 95% confidence intervals for the fitted functions are displayed as shaded regions. Red, solid—I2KI; blue, dashed—PTA; purple, dotted—PMA.

In order to compare the effects of the three contrast agents, the normalised intensity at 1 mm into each sample was measured after each scan. Since each contrast agent was used to stain four different samples there were four measurements associated with each stain. A function of the form

$$I=I_{0}+\left(I_{max}-I_{0}\right)\left(1-e^{kt}\right)$$ (1)

was fitted to each data set and plotted in Fig 4. In this equation, *I0* is the initial normalised intensity, *Imax* is the maximum normalised intensity and *k* is a parameter associated with the diffusion rate.

Qualitative differences in the progression of the different contrast agents into the samples were observed. The I2KI diffused quickly into the centres of the samples and a subsequent gradual increase of intensity ensued throughout. The PTA and PMA, however, exhibited a clear diffusion front that slowly propagated into the samples (see Fig 1). To account for this difference, the I2KI-stained samples were analysed differently to those stained with PTA and PMA.

The I2KI-stained samples were analysed using a method similar to that used to analyse the diffusion of I2KI into mouse hearts in [16]. The average normalised intensity was measured throughout the depth of each I2KI-stained sample in increments of 0.1mm and recorded as a function of time. A function of the form given in Eq (1) was fitted to the data at each spatial increment. The fitted functions were used to characterise the diffusion process, and to calculate optimal staining times as a function of distance.

**Fig 5:**
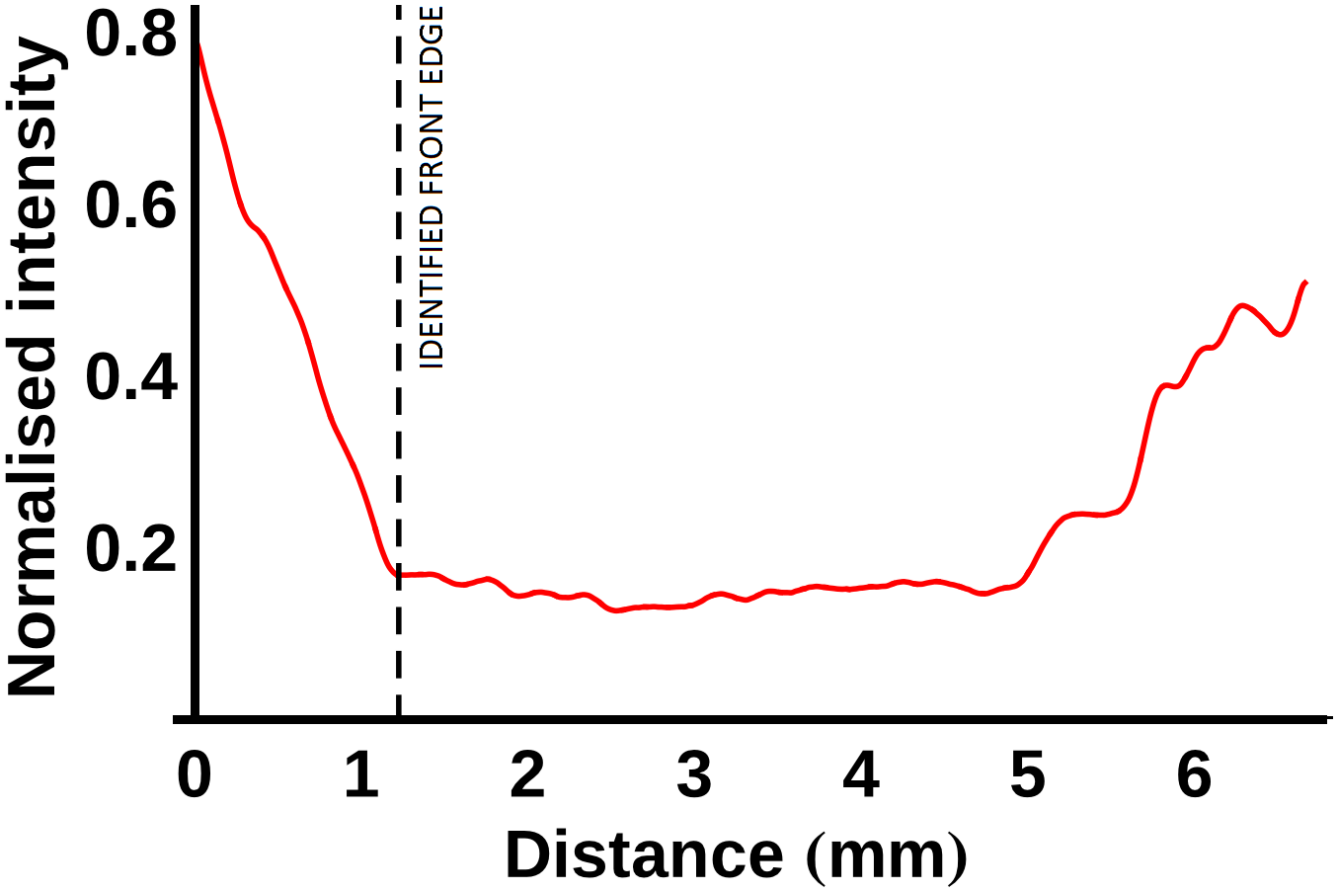
The method adopted for detecting the location of the stain front edges. The normalised intensity line profile (red line) is plotted with the distance measured from the edge of the sample. In order to detect the front edge, the first point at which the normalised intensity gradient was less than -0.2 was determined. The front edge was identified as the next point towards the sample centre at which the absolute value of the normalised intensity gradient was approximately zero (<0.01).

In order to analyse the samples stained with PTA and PMA, instead of applying the process described above, the location of the stain front was calculated using a method similar to that used to determine the location of the sample edge: 1) moving away from the previously determined edge towards the centre of a sample, the first point in the filtered data with a normalised intensity gradient less than -0.2 was determined, 2) the next point at which the absolute value of the normalised intensity gradient was approximately zero (<0.01) was identified as the edge of the stain front. This process is illustrated in Fig 5 for the PTA-stained ACL (after 1 day of staining). The average location of the staining front was calculated as a function of time and used to calculate optimal staining times as a function of distance for samples stained with PTA and PMA.

Finally, in order to measure the stain-induced shrinkage of the samples, the cross-sectional area of each sample was segmented and measured via thresholding after each scan. Additionally, 3D renderings of the samples were compared to qualitatively observe the deformation due to over-staining.

The reconstructed transverse slices were viewed, and line profiles were obtained, in *ImageJ 1*.*49m* (http://rsbweb.nih.gov/ij/). The segmentation, cross-sectional area calculations and 3D renderings were carried out in the Avizo 9.0 software environment (Visualization Sciences Group, Bordeaux (VSG), France). The differences between the line profile data sets were analysed for statistical significance using two-way analysis of variance (ANOVA) and Tukey’s post hoc test in *Mathematica 9*.*0* (Wolfram Research, Inc., Champaign, Illinois, 2012).

## Results

Two qualitative differences between the three contrast agents can be observed in Fig 4 using the fitted values of the parameters from Eq 1, which are given in Table 1. Firstly, according to the predicted values of *Imax*, it can be seen that PTA provided the greatest increase in intensity at a depth of 1mm into the samples, followed by PMA and I2KI, which give similar intensity increases. ANOVA revealed that there is a statistically significant difference in intensity between days (p<0.001) and stain types (p = 0.01). Tukey’s post-hoc test found I2KI and PTA to be significantly different at the 5% level and PTA and PMA to be significantly different at the 10% level. Secondly, the final column of Table 1 displays the normalised intensity after 4 days of staining as a percentage of its maximum predicted intensity using the values in the first three columns. It shows that by this time, the I2KI- and PMA-stained samples have gotten closer to their maximum intensities than the PTA-stained samples.

**Table 1:** The values of *I0*, *Imax*, and *k* used to fit Eq 1 to the normalised intensity profiles of the I2KI-, PTA- and PMA-stained samples as a function of time, along with the normalised intensity after 4 days of staining as a percentage of *Imax*.

Contrast agent	*I0*	*Imax*	*k* (days^-1^)	*I*|t = 4/*Imax*
I2KI	0.065	0.357	1.108	99%
PTA	0.068	0.648	0.489	87%
PMA	0.073	0.401	0.920	98%

**Fig 6:**
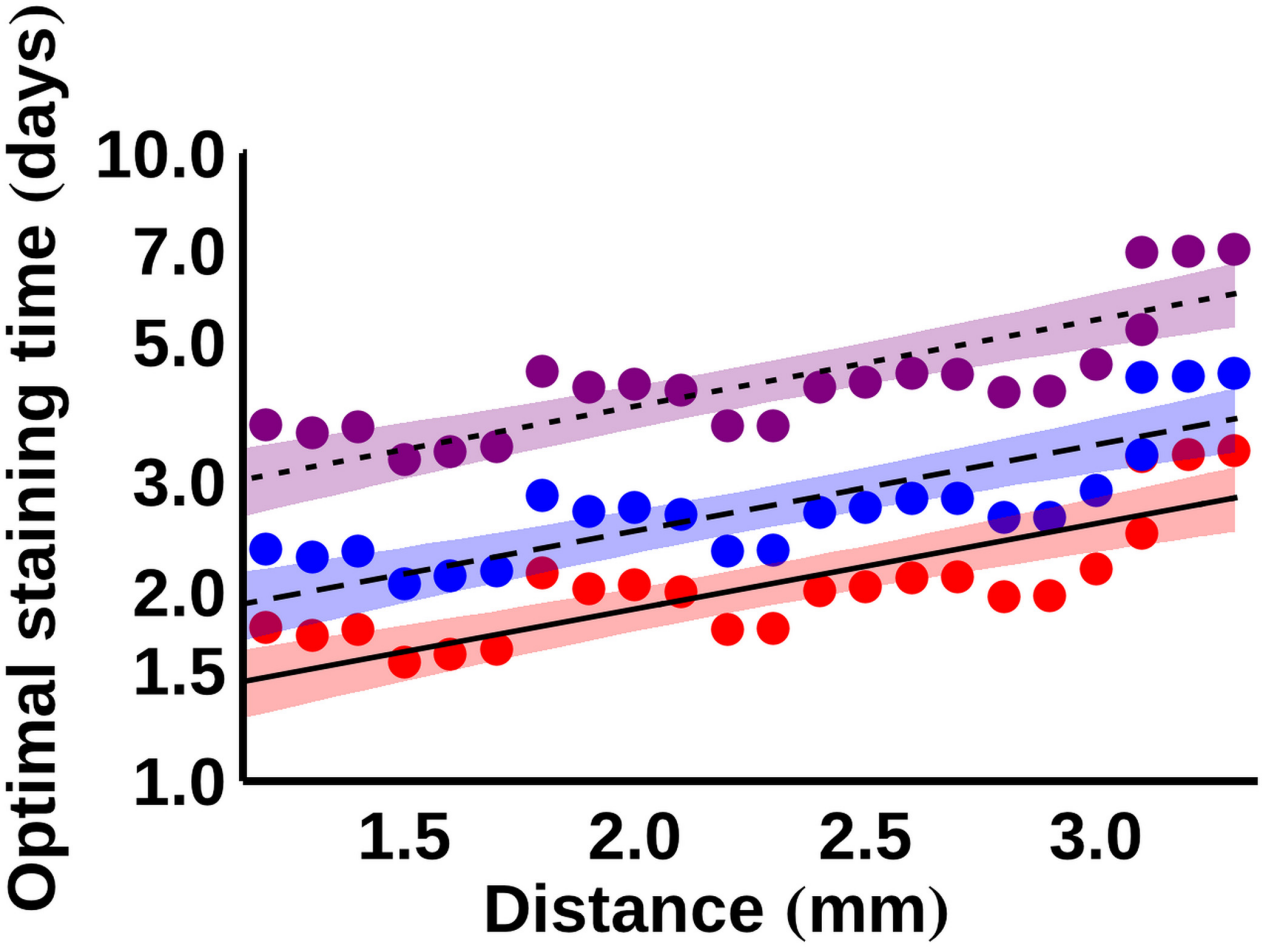
Semi-logarithmic plot of the required staining times for different levels of I2KI saturation. The curves are fitted according to Eq 2, with the fitted coefficients given in Table 2. 95% confidence intervals for the fitted functions are displayed as shaded regions. Red, solid– 90% saturation; blue, dashed– 95% saturation; purple, dotted– 99% saturation.

As mentioned above, for the I2KI-stained samples, a function of the form given in Eq 1 was fitted to the average normalised intensity data at every point at increments of 0.1mm into the samples. To determine optimal staining times for I2KI, the times at which the intensity reached 90%, 95% and 99% of its maximum value at each spatial increment were calculated using the fitted functions. Having measured these quantities for all values of *x* (the depth into the samples), an equation of the form

$$t_{stain}=Ae^{cx}$$ (2)

was fitted to the data, where *tstain* is the required staining time, and *A* and *c* are constants that take a different value for each of the three saturation levels. Fig 6 shows the result of this fitting process in the form of a semi-logarithmic plot. The fitted values of *A* and *c* are given in Table 2. The fitted equations can be used to predict the optimal staining time for a given saturation level at a certain depth; for example, to achieve 90% saturation at a depth of 1.5mm is predicted to take 1.6 days, whereas to achieve the same saturation at a depth of 3mm is predicted to take 2.6 days.

**Table 2:** The values of *A* and *c* used to fit Eq 2 to the saturation times measured using Eq 1 for various saturation levels.

Saturation level	*A* (days)	*c* (mm^-1^)
90%	1.007	0.313
95%	1.336	0.315
99%	2.100	0.317

**Fig 7:**
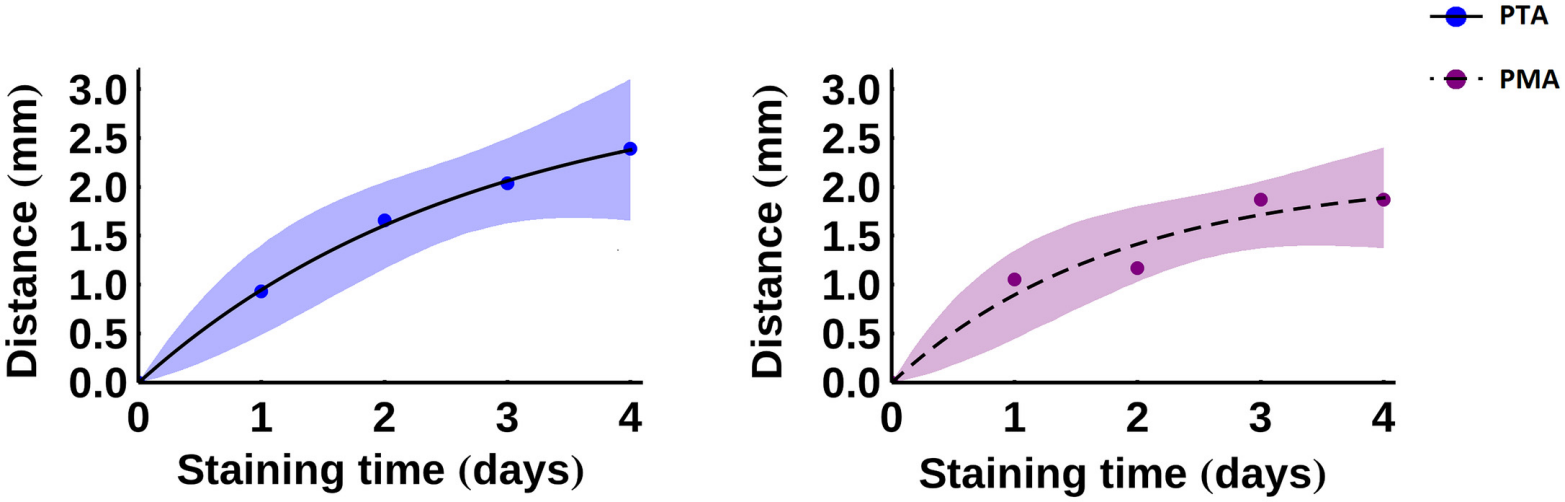
The location of the stain front as a function of time. The curves are fitted according to Eq 3, with the fitted coefficients given in Table 3. 95% confidence intervals for the fitted functions are displayed as shaded regions. Left: blue, solid—PTA; right: purple, dashed—PMA.

For the PTA- and PMA-stained samples, the location of the stain front was calculated as a function of time and a function of the form

$$x=C\left(1-e^{-Kt}\right)$$ (3)

was fitted to the data, where *C* and *K* are the fitting constants. The resulting fit is plotted in Fig 7 and the fitted values of *C* and *K* are given in Table 3. There is a statistically significant difference between days (p = 0.001), but no statistically significant difference between the two stains (p = 0.38) as determined by two-way ANOVA.

**Table 3:** The values of *C* and K used to fit Eq 3 to the front propagation data.

Contrast agent	*C* (mm)	*D* (days^-1^)
PTA	3.094	0.367
PMA	2.056	0.607

**Fig 8:**
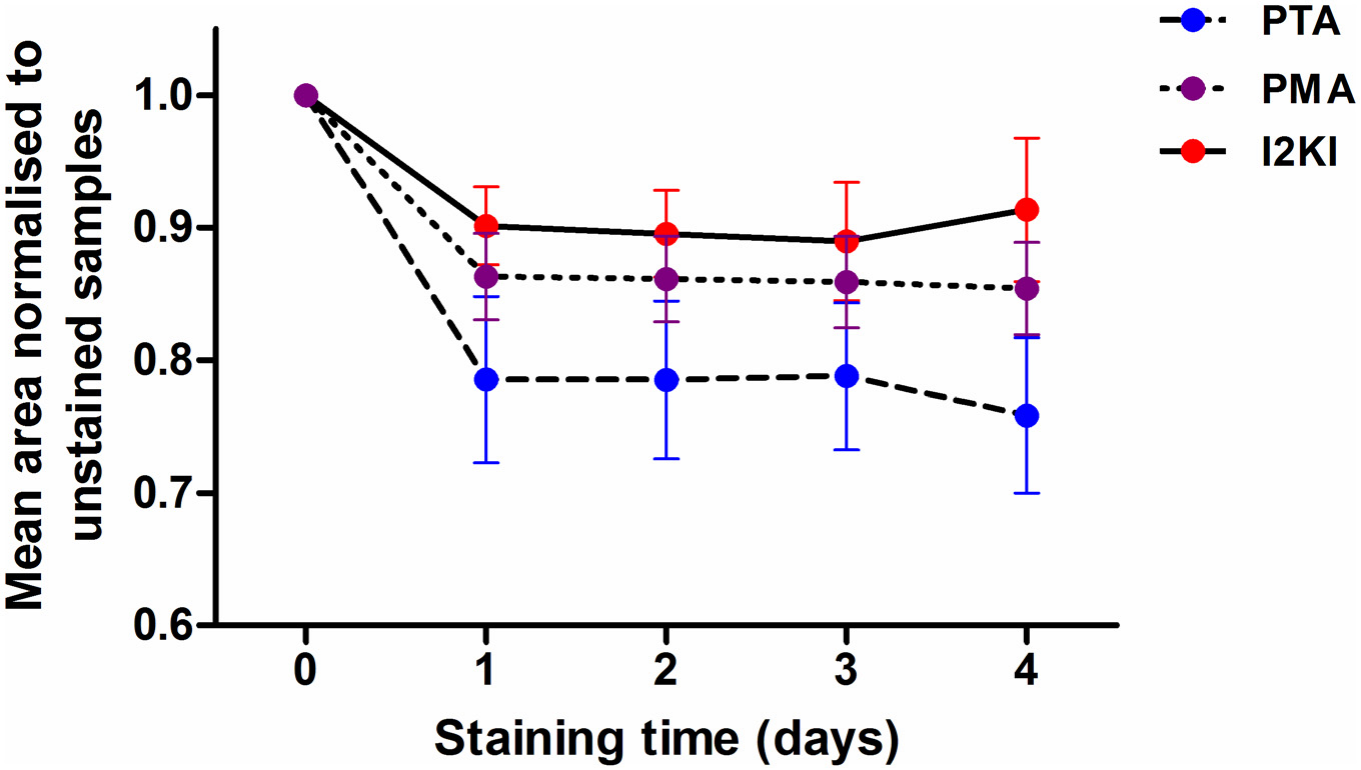
Mean, normalised cross-sectional areas of samples stained with each type of contrast agent plotted as a function of time. The cross-sectional areas of each sample were normalised to the unstained samples. The bars show ± standard error in the experimental data.

**Fig 9:**
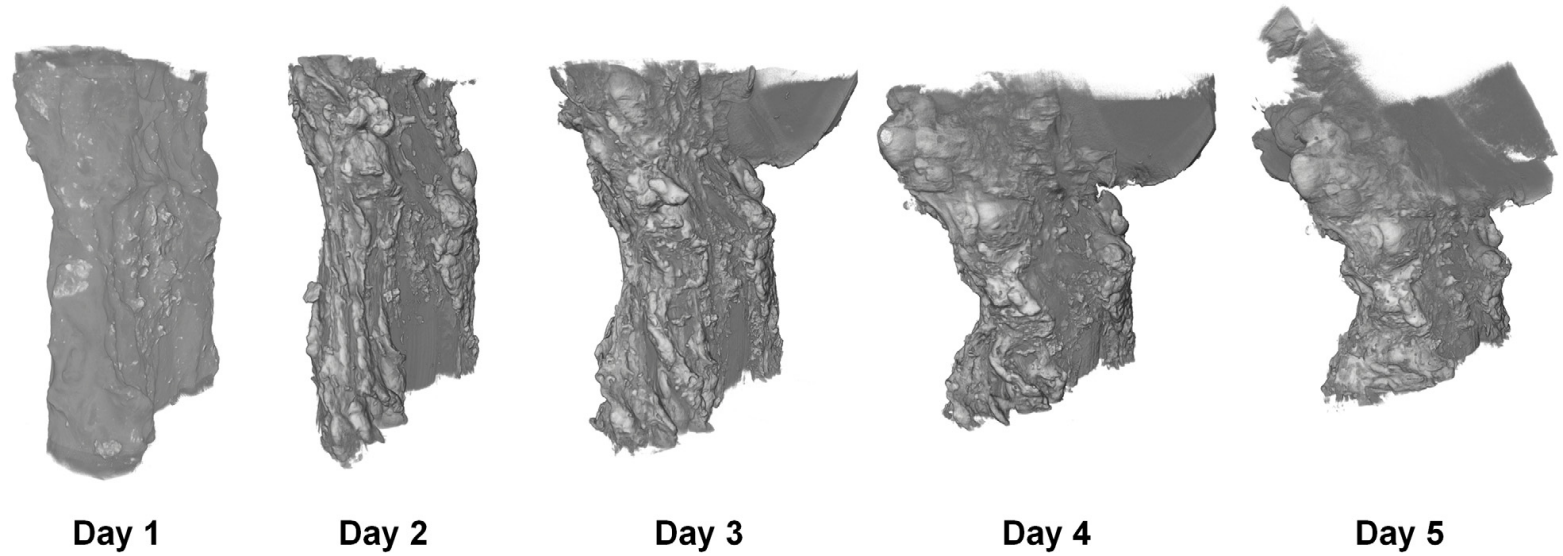
3D renderings of I2KI-stained PT after each day of staining.

The average normalised cross-sectional areas of the ligaments stained with each contrast agent are plotted as a function of time in Fig 8. The normalised value was calculated by dividing the cross-sectional area in a given sample by its initial cross-sectional area (prior to any staining). PTA caused the greatest sample shrinkage, followed by PMA, then I2KI. The shrinkage was considerable, ranging from an average of around 10% in the I2KI-stained samples to over 20% in the PTA-stained samples on average. The majority of the shrinkage occurred in the first day of staining, and the measured cross-sectional areas remained fairly constant thereafter on average. There was a statistically significant difference in areas between days (p<0.001) and stain types (p = 0.001), as determined by a two-way ANOVA. Tukey’s post-hoc test found I2KI and PTA to be significantly different at the 1% level and PTA and PMA to be significantly different at the 5% level. In addition to shrinkage, further 3D deformation took place, as illustrated in Fig 9, which was most pronounced in the I2KI-stained PT sample. This figure shows how as the sample shrinks, it begins to bend backwards upon itself—increased curvature is visible on the left side of each volume rendering as time progresses. This indicates that the stain-induced shrinkage in this ligament appears to be inhomogeneous with the left side (as viewed in Fig 9) shrinking more than the right.

## Discussion

Over the time-scales considered in this study, I2KI diffusion appeared to be qualitatively different to PTA and PMA diffusion—I2KI reached the centre of the samples rapidly, whereas PTA and PMA diffused with a clear propagation front. It is possible that I2KI also diffuses with a clear front but that this front propagates quickly enough to reach the centre of all the samples after one day of staining. Alternatively, the intensity gradient may simply be much gentler in an I2KI-stained sample, so that a front cannot be identified. To fully understand this diffusion behaviour, a series of scans with a higher time resolution than that used in this study is required.

Figs 6 and 7 can be used to estimate the amount of time that a porcine ligament sample requires in a given stain in order to achieve adequate contrast enhancement. For example, for an I2KI-stained sample with a radius of 2mm to achieve an intensity of 90% of its maximum potential value, it is estimated that a diffusion time of between approximately 1.7 and 2.0 days is required, with 95% confidence. In contrast, for the PTA stain to reach a similar depth it is estimated that a diffusion time of at least 1.8 days is necessary, and it could take longer than 4 days, with 95% confidence. PMA staining of such a sample is predicted to take at least 2.6 days, with 95% confidence. Considering the deformation that occurs in samples stained for such time periods, the size of ligaments that can be effectively scanned via contrast-agent-enhanced XCT is limited. Even if sample shrinkage is ignored, the fitted values of *C* predict an average upper sample radius limit of around 3.1 mm (95% confidence interval: 0.02 mm–5.2 mm) when using PTA and around 2.1 mm (95% confidence interval: 0.9 mm– 3.2 mm) when using PMA. These limits make the use of these contrast agents unviable for many large (e.g. porcine) ligaments/tendons.

It is noted that the optimal staining times predicted by Butters *et al*. [16] for mouse hearts are similar to those estimated here. In that study, the time required for I2KI to achieve an intensity of 90% of its maximum potential value at a depth of 2mm was predicted to be just over 45 hours. This may indicate that the differences in tissue type, contrast agent concentration (3.75% w/v in [16], compared to 10% w/v here) and contrast agent solvent (10% v/v formalin in [16], compared to water here) had little effect on the required diffusion times and that the contrast agent type is the most important factor. However, the possibility that the differences in these factors interacted to coincidentally produce similar results cannot be ruled out.

The sample shrinkage displayed in Fig 8 illustrates that contrast agents should be used with caution in biological soft tissues. Given the measured shrinkages of 10–25%, it is questionable how reliable quantitative measurements of specific features in a sample can be, especially since the shrinkage appears to be inhomogeneous, as evidenced by Fig 9. Whilst the shrinkage was much less pronounced after the first day of staining, the clear deformation displayed in Fig 9 emphasises the need to minimise staining times, even if one is prepared to accept a certain amount of shrinkage.

## Limitations

The edges of the samples were detected by finding the location of maximum intensity gradient and defining the next point towards the centre of the sample where the intensity gradient was approximately zero. This method was chosen as it was used by Butters *et al*. in a recent paper [16] and therefore allows a direct comparison between the two studies. If the edges in this study had instead been defined as the points of maximum intensity gradient then they would have been further from the centre by an average of 1.1% of the total calculated width of the sample and a maximum of 3.7% across all sixty line profiles. Therefore, all reported depths in this paper may be considered to be a slight underestimation.

It is noted that streaking artefacts are present in many of the tomographic slices (see Fig 1). These may be the result of a slight misalignment between projections taken from opposing angles (i.e. 0° and 180°, or 90° and 270°). To reduce scanning times, the samples were allowed to rotate continuously instead of being made to stop before each projection was taken; therefore, each projection will have taken place over a small angular range rather than a single discrete angle, which may have caused the slight misalignment. The resulting artefacts could thus be reduced at the expense of increasing acquisition time by forcing the samples to stop rotating between each projection. As a result of these artefacts, it was not possible to automatically segment all of the sample volumes in 3D. Therefore, cross-sectional areas of tomographic slices that *could* be segmented were used to calculate estimates of sample shrinkage as displayed in Fig 8; however, these measurements do not take account of 3D effects. It is also possible that some of the observed shrinkage in this study was a result of the samples drying during scanning. An attempt to minimise this source of shrinkage was made by including a reservoir of phosphate-buffered saline solution in the scanning vessel; however, further work would be necessary to quantify the level of shrinkage that is caused by sample drying.

The deformation observed in iodine-stained soft tissue samples has been attributed to the hypertonic nature of iodine [25], but it may be possible to largely eliminate this deformation by introducing a hydrogel stabilisation step prior to contrast agent immersion [25]. It would be of interest to investigate whether a similar preparation procedure would reduce or eliminate shrinkage in PTA- and PMA-stained samples.

The focus of this study was to determine optimal diffusion times using contrast agents under constant conditions; however, many of the factors that may affect these diffusion times were not considered. A list of these factors and the values used in this study is given in Table 4. Further studies would be required to determine the relative effects of these factors. Whilst it has been shown that I2KI concentration significantly impacts upon scan results [10–12], to the authors’ knowledge, no such studies have been carried out on the effect of PTA or PMA concentration.

**Table 4:** Summary of factors that may affect optimal diffusion times.

Factor	Type/value used in the present study
Tissue type	Porcine ligaments and tendons
Fixative	10% v/v formalin
Contrast agent	I2KI, PTA and PMA
Contrast agent concentration	10% w/v
Contrast agent solvent	Water
Temperature	Room temperature
Pressure	Atmospheric pressure

## Conclusions

The results of this study indicate that contrast enhancement in XCT of porcine ligaments and tendons is highly dependent on diffusion times. The optimal staining times plotted in Figs 6 and 7 can be used as a guide of how long samples of a given size should be immersed in contrast agents. Due to the significant sample shrinkage and the deformation that takes place during prolonged staining, it is recommended that contrast-enhanced XCT is primarily used on smaller ligament/tendon samples.
